# *Notes from the Field:* Dengue Outbreak — Peru, 2023

**DOI:** 10.15585/mmwr.mm7304a4

**Published:** 2024-02-01

**Authors:** César V. Munayco, Betsabet Yadira Valderrama Rosales, Susan Yanett Mateo Lizarbe, Carmen Rosa Yon Fabian, Ricardo Peña Sánchez, César Henry Vásquez Sánchez, Maria Paquita García, Carlos Padilla-Rojas, Victor Suárez, Liliana Sánchez-González, Forrest K. Jones, Luciana Kohatsu, Laura E. Adams, Juliette Morgan, Gabriela Paz-Bailey

**Affiliations:** ^1^Centro Nacional de Epidemiología, Prevención y Control de Enfermedades, Peru Ministry of Health, Lima Peru; ^2^Ministry of Health, Lima Peru; ^3^Instituto Nacional de Salud, Ministry of Health, Lima, Peru; ^4^Division of Vector-Borne Diseases, National Center for Emerging and Zoonotic Infectious Diseases, CDC; ^5^Epidemic Intelligence Service, CDC; ^6^South America Regional Office, CDC, Brasilia, Brazil.

**Summary**

**What is already known about this topic?**

Dengue, a mosquitoborne viral disease, is endemic to Peru, with the annual number of cases ranging between 4,698 and 68,290 from 2017 to 2022.

**What is added by this report?**

In March 2023, a sharp increase in dengue cases in Peru occurred. In the first 30 weeks of the year, 222,620 cases (exceeding the previous 5-year average by a factor of approximately 10) and 381 dengue-associated deaths were reported. The Lima metropolitan area experienced a substantially higher incidence compared with historical levels, when few locally acquired cases were observed.

**What are the implications for public health practice?**

Dengue outbreaks can be abrupt and can strain health care systems, necessitating rapid outbreak detection in addition to intensive preparedness and efforts to strengthen response capacity at the primary care level.

Dengue, a mosquitoborne viral disease, is endemic to Peru, with highest seasonal transmission usually occurring between November and May (*1*). All four dengue viruses (DENV 1–4) have circulated in Peru, most commonly DENV-1 and DENV-2 (*2*). Historically, departments (the first level administrative subdivision) in the north have reported the highest dengue incidence, whereas incidence in the Lima metropolitan area on the central Pacific coast (population approximately 11 million) has been low.

## Epidemiologic Findings

In March 2023, the mean weekly number of dengue cases in Peru increased sharply (Figure), from 2,182 during epidemiologic weeks 1–10 (corresponding to January 1–March 11) to 8,787 during weeks 11–20 (March 12–May 20). As of the end of week 30 (July 29), the 222,620 cases in 2023 were approximately 10 times the average number during the same period during the previous 5 years (21,841 cases) and 3.5 times the number during the same period in 2017 (64,431 cases), the year of the largest previous national dengue outbreak. A nationwide epidemiologic alert to notify health care providers of the risk of dengue outbreaks was issued on April 21. CDC employees were deployed to Peru at the end of May to collaborate on the outbreak investigation. This activity was reviewed by CDC, deemed not research, and was conducted consistent with applicable federal law and CDC policy.*

**FIGURE:**
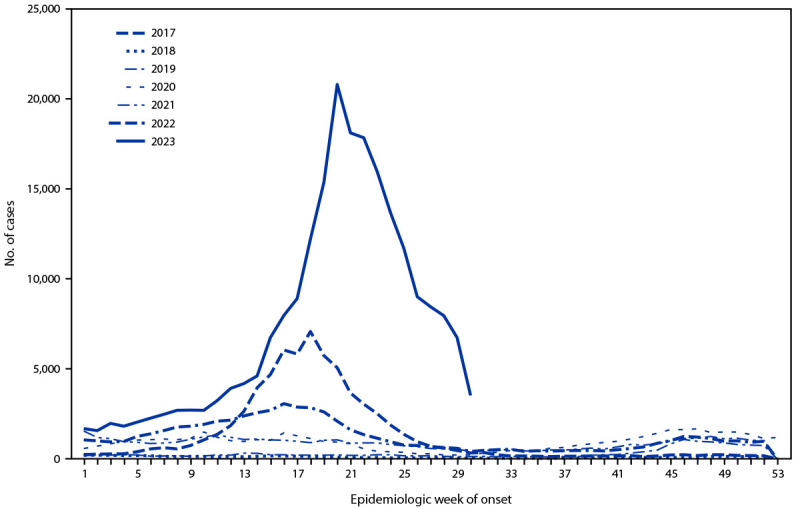
Weekly number of dengue cases reported nationwide,* by epidemiologic week^†^ — Peru, January 1, 2017–July 29, 2023 * Population = 34 million. ^†^ Epidemiologic weeks begin on Sunday and end on Saturday. 2023 data are shown through epidemiologic week 30.

During January 1–July 29, a total of 83,254 probable^†^ and 139,366 confirmed^§^ dengue cases were reported, making this the largest dengue outbreak on record in Peru. Several departments^¶^ with the highest numbers of cases (located in coastal northwestern Peru), including Piura (67,697), Lambayeque (28,235), and La Libertad (20,289) (Supplementary Table, https://stacks.cdc.gov/view/cdc/147148), also reported high dengue incidence during the 2017 outbreak and were affected by extreme rainfall in early March 2023 related to Cyclone Yaku.** Case counts in Lima (32,009) were much higher than those reported during previous years, including in neighborhoods that have not historically reported dengue cases. The highest age-specific incidence (807 cases per 100,000 population) was reported among persons aged 12–17 years; 55% of cases occurred in females.

## Mortality

Overall, 381 dengue-related deaths were reported (case fatality ratio [CFR] = 0.17%). More than one half of all deaths (204, 54%) occurred among persons aged ≥60 years, who also experienced the highest CFR (0.90%), and nearly one third of dengue-related deaths (109, 29%) occurred in persons aged 30–59 years (CFR = 0.13%). Persons aged <30 years with dengue experienced the fewest number of deaths (68, 18%) and the lowest CFR (0.06%).

The largest number of deaths occurred in Piura (130 deaths, CFR = 0.19%), followed by Lambayeque (115, CFR = 0.41%), and Ica (52, CFR = 0.32%). Dengue-related deaths were reported in 16 (64%) of 25 jurisdictions.

## Diagnostic Testing

Molecular and serologic diagnostic testing, including real-time reverse transcription–polymerase chain reaction nonstructural protein 1 antigen, and immunoglobulin M enzyme-linked immunosorbent assay testing were conducted through a network of 49 public health laboratories. These laboratories conducted more than 200,000 tests in 2023. Among 14,462 cases with DENV serotype available in 2023, DENV-2 was the most common serotype identified (7,105, 49%), followed by DENV-1 (7,038, 49%), and DENV-3 (319, 2%).^††^

## Preliminary Conclusions and Actions

The Ministry of Health of Peru, in collaboration with regional health offices and international partners, implemented a broad, integrated surveillance and response strategy, including increased targeted larvicidal treatments of standing water and insecticide spraying in affected neighborhoods. Clinical surveillance units with dedicated personnel with training in dengue clinical management were established in outbreak areas, and hospitals implemented triage tents for febrile patients; in-person and online trainings were available to clinicians nationwide.

Dengue is a growing health threat globally, with multiple factors potentially contributing to the increasing incidence and expansion into new areas including rapid urbanization, increased travel, and climate change (*3*). Dengue outbreaks can be abrupt and strain health care systems, requiring rapid recognition of transmission and intensive preparedness and efforts to strengthen response capacity at the primary care level. Additional interventions and resources, including vaccines and effective and scalable vector control methods, are increasingly critical to reducing dengue morbidity and mortality (*4*). Public health agencies can prepare for and respond to dengue outbreaks by evaluating and supporting implementation of effective vector control methods and vaccines, strengthening dengue surveillance, and reinforcing clinical management training to improve patient outcomes.

## Footnotes

* 45 C.F.R. part 46, 21 C.F.R. part 56; 42 U.S.C. Sect. 241(d); 5 U.S.C. Sect. 552a; 44 U.S.C. Sect. 3501 et seq.

† Probable cases are defined as a febrile illness lasting ≤7 days in a person who resides in or has visited areas with dengue virus transmission or *Aedes aegypti* infestation within 14 days before the onset of symptoms and who has at least two of the following signs and symptoms: ocular or retro-orbital pain, myalgia, headache, arthralgia, lower back pain, rash, and nausea or vomiting. In addition, probable cases have no laboratory test result or epidemiologic linkage to other dengue cases.

§ Confirmed cases have the same symptom and exposure criteria as do probable cases but have received at least one positive result from one or more of the following tests: 1) virus isolation by cell culture, 2) real-time quantitative reverse transcription–polymerase chain reaction, 3) enzyme-linked immunosorbent assay (ELISA) nonstructural protein 1 antigen, 4) detection of immunoglobulin M (IgM) antibodies for dengue in a single sample by ELISA (for areas with endemic dengue), and 5) IgM evidence of seroconversion in paired samples (in areas where there is no dengue transmission and cases must have an epidemiologic investigation).

¶
https://www.dge.gob.pe/sala-situacional-dengue/#grafico01

**
https://www.reuters.com/business/environment/heavy-rainfall-peru-slammed-infrastructure-losses-seen-323-mln-2023-03-22/

†† https://app.powerbi.com/view?r=eyJrIjoiOTQ0MzllOTItNWNkNC00MzE3LWJiM2QtZGUyYjU0NWFjYjUyIiwidCI6ImI0NzYxY2VlLTlkYWQtNDc3MS05ZjQ3LTVmYjc4Y2MxYjRhYSIsImMiOjR9&pageName=ReportSection73939390533a7a82da04 (accessed Jan 26, 2024).
